# Increased Arterial Stiffness as a Predictor for Onset and Progression of Diabetic Retinopathy in Type 2 Diabetes Mellitus

**DOI:** 10.1155/2021/9124656

**Published:** 2021-09-23

**Authors:** Yaxin An, Yuxian Yang, Bin Cao, Huan Dong, Aihua Li, Wenying Zhao, Jing Ke, Dong Zhao

**Affiliations:** ^1^Center for Endocrine Metabolism and Immune Diseases, Beijing Luhe Hospital, Capital Medical University, Beijing 101149, China; ^2^Beijing Key Laboratory of Diabetes Research and Care, Beijing 101149, China

## Abstract

**Introduction:**

Brachial–ankle pulse wave velocity (baPWV), an indicator of arterial stiffness, has been demonstrated to be associated with type 2 diabetes mellitus (T2DM) and its vascular complications. This study was aimed at investigating the correlations of baPWV with both the presence and severity of diabetic retinopathy (DR) at baseline and at exploring the predictive role of baPWV in the new onset/progression of DR in the follow-up analysis.

**Methods:**

The prospective cohort study recruited 2,473 Chinese patients with T2DM, of whom 663 participants were finally included in the follow-up analysis. The presence and grading of DR were performed by the modified Early Treatment Diabetic Retinopathy Study. Uni- or multivariate linear and logistic regression models and Cox proportional-hazards regression analysis were conducted.

**Results:**

Of 2,473 patients with T2DM at baseline, 734 individuals were assessed to have DR and further categorized into 630 with non-sight-threatening DR (NSTDR) and 104 with STDR. In addition to the positive relationship between increased baPWV and the presence of DR, multinominal logistic regression analysis revealed that higher tertiles of baPWV were significantly related to the NSTDR (T2: OR = 1.62 (1.22, 2.15), *p* < 0.001, and T3: OR = 2.58 (1.86, 3.58), *p* < 0.001) and STDR group (T3: OR = 3.87 (1.87, 8.02), *p* < 0.001). During a follow-up (mean period of 16.4 months), 111 participants had new onset/progression of DR. The cox regressions showed that high baseline baPWV was correlated with increased risk of development/progression of DR (HR = 2.24, 95% CI (1.24, 4.03), *p* = 0.007, for T2 baPWV and HR = 2.90, 95% CI (1.49, 5.64), *p* = 0.002, for T3 baPWV) after adjustments for multiple factors.

**Conclusions:**

Our results demonstrated that baseline baPWV might be an independent predictor in new onset/worsening of DR, suggesting that increased arterial stiffness might be involved in the development of DR. Follow-up studies with a longer duration are needed.

## 1. Introduction

Type 2 diabetes mellitus (T2DM) is a global pandemic posing an enormous health and economic burden to both individuals and societies [1]. As one of the most common microvascular complications of T2DM, diabetic retinopathy (DR) affects over one-third of individuals with diabetes worldwide [2] and approximately one-fifth in China [3]. Furthermore, DR, which primarily contributes to vision impairment and irreversible blindness among working-age adults, is associated with higher morbidity and disability [4, 5].

Existing evidence has suggested the link between DR and diabetic macrovascular complications, specifically atherosclerotic cardiovascular disease [6–8]. Arterial stiffness (AS), closely related to aging and atherosclerosis, reflects arterial structure and function. Pulse wave velocity (PWV), widely applied as a noninvasive index of AS, is an established risk factor of cardiovascular events or all-cause mortality in different populations [9–11], involving individuals with T2DM [12, 13]. PWV, which indirectly measures the time necessary for pulse waves to cross an artery of a given distance, can be evaluated through two regional arterial sites, such as carotid-femoral PWV (cfPWV, an index of central AS or aortic stiffness) and brachial-ankle PWV (baPWV, a marker of peripheral AS) [14].

Subjects with diabetes have exhibited worsening AS [15, 16]. Evidence for positive associations of increased PWV with the presence [14, 17–19] and the severity of DR [20–22] has been accumulating from cross-sectional studies. However, the predictive role of AS in the development of DR by longitudinal studies has been less studied. Therefore, we conducted a prospective cohort study in Chinese patients with T2DM to investigate the correlations of baPWV with both the presence and severity of DR at baseline and to explore the role of baPWV in the development or progression of DR in the follow-up analysis.

## 2. Materials and Methods

### 2.1. Study Population

A total of 2,473 Chinese patients with T2DM, admitted to the outpatient department or hospitalized at the Center for Endocrine Metabolism and Immune Disease of Beijing Luhe Hospital, Capital Medical University, were finally enrolled in this study. All subjects participated in the program of National Metabolic Management Center (MMC) [23]. Of note, most of the patients were followed up regularly according to the protocols of MMC for professional management and guidance. The diagnosis of T2DM was defined based on World Health Organization criteria in 1999 [24], as described in our previous study [25]. Exclusion criteria were outlined as below: other types of diabetes, history of malignant tumors, with acute coronary syndrome, history of cerebrovascular disease, with peripheral artery disease defined as ankle-brachial index (ABI) < 0.9, chronic hepatic cirrhosis, renal insufficiency with an estimated glomerular filtration rate (eGFR) less than 30 mL/min/1.73 m^2^, with contraindication or not willing to undergo fundus photography, other ophthalmic lesions, or impossible reading of fundus images, with missing data regarding baPWV. Additionally, 663 patients had been followed up with a mean duration of 16.4 ± 6.5 months. The other patients enrolled at baseline were excluded for the follow-up analysis primarily due to a short duration of follow-up or lack of data concerning fundus photography at the most recent follow-up. The protocol for this study was approved by the Ethical Review Committee of Beijing Luhe Hospital. Written informed consent was signed from all subjects at the baseline enrollment in the study.

### 2.2. The Assessment of DR and Subgroups of the Study Population

All patients were evaluated for DR at baseline and possibly at follow-ups annually or biannually, as appropriate. A nonmydriatic fundus camera (TRC-NW400, Topcon, Tokyo, Japan) was employed by two trained specialists to capture four 45-degree color fundus images centered on the optic papilla and macula in both eyes. In accordance with the modified criteria of Early Treatment of Diabetic Retinopathy Study [26], the presence and grading of DR were assessed by the same personnel, blinded to the patient's information. At baseline, all the patients were divided into non-DR (NDR) and DR groups. If present, each eligible eye was graded as mild, moderate, severe nonproliferative DR (NPDR), and proliferative DR (PDR) [26]. According to the American Diabetes Association (ADA) criteria [27], patients with DR were reclassified into non-sight-threatening DR (NSTDR), including mild and moderate NPDR, and sight-threatening DR (STDR), comprising severe NPDR (a precursor of PDR) and PDR. When there was a difference in the interpretations of DR grading between two eyes, the severity was recorded from the worse-affected eye. For those who underwent retinal examinations at follow-ups, development/progression of DR was defined as either new occurrence or deterioration of the baseline DR grading in each eye.

### 2.3. Measurement of Peripheral Arterial Stiffness

Following a rest in the supine position for at least 5 minutes, patients underwent measurement of baPWV by using an automated arteriosclerosis detection device (Model BP-203 RPE III, Omron Health & Medical (China) Co., Ltd.). The device recorded pulse wave data from sensors in the four cuffs applied on brachial and tibial arteries from the upper limbs and ankles. The average of baPWV values from both sides was recorded and used for further analysis.

### 2.4. Clinical and Laboratory Measurements

Based on the MMC protocol, demographic and clinical data, including age, gender, duration of T2DM, smoking and alcohol status, comorbidities, detailed medical history, and medications, were collected from each subject at baseline and possible follow-ups. In addition, height and body weight were assessed to calculate body mass index (BMI). Blood pressure (BP) was measured, and hypertension was diagnosed in patients who had systolic BP ≥ 140 mmHg and/or diastolic BP ≥ 90 mmHg or were on antihypertensive agents. Mean arterial pressure (MAP) was calculated as the sum of diastolic BP and one-third of pulse pressure. Hyperlipidemia was defined as triglycerides (TG) > 1.7 mmol/L or total cholesterol (TC) > 5.17 mmol/L or low-density lipoprotein cholesterol (LDL − c) > 3.36 mmol/L or current usage of lipid-lowering medications. The urine albumin-creatinine ratio (UACR) was calculated. Albuminuria, as a categorical variable, was classified as normoalbuminuria (<30 mg/g), microalbuminuria (30-300 mg/g), and macroalbuminuria (≥300 mg/g) by using UACR levels. Current smoking was ascertained as self-reported smoking daily or almost daily. Alcohol consumption was reported as current alcohol drinking at least once a week or almost per week.

After an overnight fast, venous blood samples were drawn in the morning to determine routine laboratory parameters, including fasting serum glucose, glycosylated hemoglobin (HbA1c), fasting serum C-peptide, serum concentrations of creatinine, blood urea nitrogen, uric acid, TC, TG, LDL-c, high-density lipoprotein cholesterol (HDL-c), aspartate aminotransferase (AST), and alanine aminotransferase (ALT). The eGFR was computed according to the Chronic Kidney Disease-Epidemiology Collaboration (CKD-EPI) equation [28].

### 2.5. Statistical Analysis

Parametric continuous variables were expressed as mean ± standard deviation, and nonparametric data were shown as median and interquartile ranges. Categorical variables were presented as numbers and proportions. A two-tailed *t*-test for continuous data was employed for comparisons between two groups. Differences among three groups were analyzed by one-way analysis of variance or Wilcoxon test, as appropriate. When necessary, Tukey's multiple comparisons or Stell-Dwass test were used for post hoc analysis. Comparisons among categorical variables were performed by a chi-square test. Patients enrolled at baseline were additionally separated into three groups according to tertiles of baPWV, with 1^st^ tertile (T1, baPWV < 13.67 m/s), 2^nd^ tertile (T2, 13.67 ≤ baPWV < 16.16 m/s), and 3^rd^ tertile (T3, baPWV ≥ 16.16 m/s). Similarly, tertiles of baPWV (with cut-off values of 13.62 and 15.93 m/s) were also calculated among those who received follow-ups. The differences of baPWV among groups stratified by the presence or severity of DR were adjusted by multivariate linear regression. Furthermore, we performed multivariate logistic regressions to investigate the independent association of degrees of baPWV on the presence of DR at baseline. In the models, the T1 baPWV was selected as a reference category. Regarding the relationship between baPWV and the severity of DR, multinominal logistic regression analysis was performed, where the NDR group was used as a reference group. Multivariate models at baseline analysis were adjusted for potential covariates, including age, gender, BMI, current smoker, MAP, antihypertensive drugs, history of cardiovascular disease, duration of T2DM, HbA1c, TG, HDL-c, LDL-c, lipid-lowering drugs, eGFR, albuminuria, uric acid, ALT, and AST. Moreover, to explore whether baseline baPWV was independently associated with development/progression of DR, we used Cox proportional-hazards regression analysis, adjusted for age, gender, duration of T2DM, HbA1c, MAP, antihypertensive medication, hyperlipidemia, eGFR, albuminuria, and uric acid. All statistical analyses were performed by using R (version 4.1.2, http://www.R-project.org, the R Foundation). A *p* value less than 0.05 was considered to be statistically significant, and a *p* value ranging from 0.05 to 0.06 was reported as a trend.

## 3. Results

### 3.1. Baseline Characteristics of Patients with T2DM

The baseline clinical and biochemical parameters of all patients with T2DM according to tertiles of baPWV are summarized in Supplemental Table 1. Among 2,473 patients with T2DM, 734 patients (29.7%) were assessed to have DR. Those with DR were further categorized into 630 patients (25.5%) with NSTDR, including 484 (19.6%) mild and 146 (5.9%) moderate NPDR, as well as 104 (4.2%) having STDR, which consisted of 61 (2.5%) severe NPDR and 43 (1.7%) PDR.

### 3.2. baPWV and the Presence of DR

**Figure 1:**
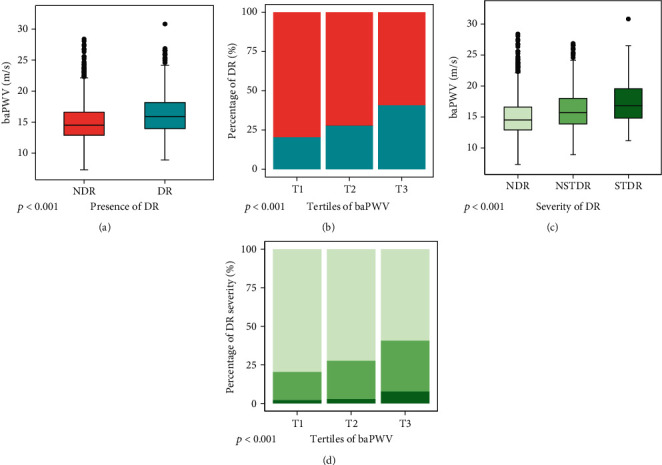
The associations of baPWV with the presence and severity of DR. Differences in baPWV according to the presence of DR (a) and severity of DR (c) were presented by boxplots. Stacked bar plots (b) showing percentages of the presence (bluish-green bar) and absence of DR (orange bar) stratified by tertiles of baPWV, while similar plots (d) displaying differences in the proportions of severe DR (dark green) and mild DR (medium green) among tertiles of baPWV.

As shown in Figure 1(a), a statistically higher value of baPWV was observed in patients with DR than in those without DR (16.29 ± 3.27 vs. 14.94 ± 2.90, *p* < 0.001, Figure 1(a)). After controlling for potential covariates, including age, gender, BMI, current smoker, MAP, antihypertensive drugs, history of cardiovascular disease, duration of T2DM, HbA1c, TG, LDL-c, HDL-c, lipid-lowering drugs, eGFR, albuminuria, uric acid, ALT, and AST, multiple linear regression analysis displayed that the difference in baPWV between the two groups remained significant (*β* = 0.87, 95% CI (0.65, 1.10), *p* < 0.001).

When all patients were categorized into three groups based on tertiles of baPWV, the prevalence of DR increased with increasing tertiles of baPWV (20.4% vs. 27.7% vs. 40.7% in T1, T2, and T3, respectively, *p* < 0.001, Supplemental Table 1 and Figure 1(b)).

In addition, Table 1 (the upper part) summarized the consistent results of uni- and multivariate logistic regression analyses, shown as odds ratios (OR) together with their 95% confidence intervals (CI). With increases in baPWV, the relative risk of having DR increased (OR = 1.59, 95% CI (1.21, 2.10), *p* = 0.001, for T2 baPWV and OR = 2.73, 95% CI (2.00, 3.74), *p* < 0.001, for T3 baPWV, Table 1).

### 3.3. baPWV and the Severity of DR

With respect to the severity of DR, patients with DR were further divided into NSTDR and STDR groups. It was observed that baPWV was significantly higher in patients with STDR compared to the NSTDR and NDR group (17.33 ± 3.64 vs. 16.12 ± 3.18 and 14.94 ± 2.90, *p* < 0.001, Figure 1(c)). Likewise, the comparisons of baPWV stratified by the DR stages, adjusted for potential confounding factors by using multiple linear regression analysis, remained significant (*β* = 0.80, 95% CI (0.57, 1.04), *p* < 0.001, in the NSTDR group and *β* = 1.31, 95% CI (0.83, 1.79), *p* < 0.001, in the STDR group).

As presented in Figure 1(d), there was a marked influence of baPWV on the proportions of DR gradings (*p* < 0.001), showing that most patients with STDR and/or NSTDR were observed in the subgroup with the highest tertile of baPWV (7.6% vs. 2.1% and 2.8%, 33.1% vs. 18.3% and 24.9%, respectively, Supplemental Table 1 and Figure 1(d)).

Similarly, the crude and adjusted models on the basis of the severity of DR are presented in Table 1 (the lower part). There was a significant relationship between increased baPWV and the NSTDR group (T2 baPWV: OR = 1.62, 95% CI (1.22, 2.15), *p* < 0.001; T3 baPWV: OR = 2.58, 95% CI (1.86, 3.58), *p* < 0.001, Table 1). However, the STDR group was only related to T3 baPWV (OR = 3.87, 95% CI (1.87, 8.02), *p* < 0.001, Table 1), showing the highest OR value (*p* = 0.310).

### 3.4. Baseline baPWV and the Development/Progression of DR

Of 2,473 patients with T2DM enrolled at baseline, 663 patients, who received regular follow-ups with a mean duration of 16.4 months and underwent the fundus photography at the most recent follow-ups, were finally included in the follow-up analysis. Each participant was evaluated whether there is a new onset or a progression in DR stages in each eye. As a result, 111 (16.7%) participants were classified in the group with development/worsening of DR. The baseline characteristics of patients included in the follow-up analysis are summarized in Table 2.

**Figure 2:**
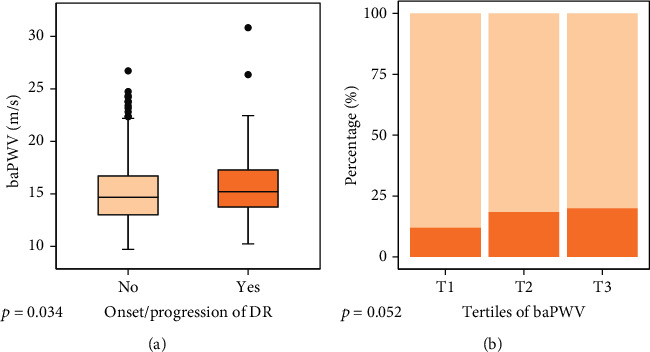
The relationships between baPWV and new onset/progression of DR: (a) boxplots showing distributions of baPWV between the group without and with new onset/progression of DR; (b) comparisons of the group with (dark orange) and without onset/progression of DR (light orange) based on the tertiles of baPWV were illustrated by stacked bar plots.

Concerning the AS, the patients with development/progression of DR exhibited statistically higher values of baseline baPWV in comparison with those without progression in DR (15.72 ± 3.27 vs. 15.08 ± 2.85, *p* = 0.034, Figure 2(a)). After adjustments for age, gender, duration of T2DM, HbA1c, MAP, antihypertensive medication, hyperlipidemia, eGFR, albuminuria, and uric acid, the baPWV was statistically different between the two groups (*β* = 0.68, 95% CI (0.26, 2.63), *p* = 0.009). According to the three groups stratified by tertiles of baseline baPWV, patients with increasing tertiles of baPWV tended to have a higher incidence of development/progression of DR (11.9% vs. 18.7% vs. 19.6%, *p* = 0.052, Table 2 and Figure 2(b)).

Moreover, we performed univariate Cox proportional-hazards regression analysis to explore the potential risk factors associated with the development/progression of DR. In addition to duration of T2DM, HbA1c, and eGFR, baPWV, as a continuous variable, was related to the development/progression of DR (HR = 1.09, 95% CI (1.03, 1.15), *p* = 0.004).

Furthermore, several multiple Cox regression models controlled for different covariates, where T1 baPWV used as a reference, were utilized to demonstrate the independent effect of baPWV degrees on the development/progression of DR (Table 3). The fully adjusted model 3 documented that increasing baPWV was correlated with a higher risk of development/progression of DR (HR = 2.24, 95% CI (1.25, 4.03), *p* = 0.007, for T2 baPWV and HR = 2.90, 95% CI (1.50, 5.64), *p* = 0.002, for T3 baPWV, Table 3) after adjustments for age, gender, duration of T2DM, HbA1c, MAP, usage of hypertensive medication, hyperlipidemia, eGFR, albuminuria, and uric acid. It suggested that patients with higher baseline baPWV were more likely to develop DR or progress in DR stages at follow-up.

## 4. Discussion

In the present study, which recruited 2,473 Chinese patients with T2DM, increased AS, indicated by a high value or an increased degree of baPWV, was positively related to the presence and the severity of DR, including the NSTDR and STDR group. The major finding of this study was that a higher baseline baPWV was independently associated with new occurrence or progression of DR in the subsequent follow-up analysis. To the best of our knowledge, this is the first longitudinal study to investigate the predictive role of baPWV in DR among Chinese populations with diagnosed T2DM.

Our results, showing that worsening peripheral AS was positively correlated with the presence of DR after adjustments for multiple confounding factors, are consistent with previous studies [17–19]. Several other reports documented a significant relationship between central AS, indicated mainly by cfPWV, and the presence of DR [14, 21]. However, the superior method regarding PWV measurements from different segments in the evaluation of DR is controversial [14, 29]. The measurement of cfPWV, assessing the stiffness of large artery, requires specialized training and complicated procedures with a long period of detection, as well as exposure of the sensitive part of the body [30, 31]. By contrast, baPWV is a more convenient and automatic measurement with good reproducibility [32], making it more appropriate for large population screening [33]. Additionally, compared to cfPWV, baPWV reflects the stiffness of the aorta and more peripheral arteries in four limbs affected by vasomotor reflex, measuring a longer distance and more muscles and elastic arteries [29]. Moreover, baPWV has been shown as a surrogate of aortic stiffness [34, 35]. BaPWV is, therefore, a favorable and widely adopted marker of AS.

With respect to the severity of DR, previous studies demonstrating a positive correlation with peripheral or central AS [20–22] were fully supported by our results, which documented that baPWV was associated with higher odds for NSTDR and STDR. Different from the other studies that mostly employed the classic categorization of DR stages, i.e., NPDR and PDR, we divided the DR group into NSTDR, comprising mild and moderate NPDR, and STDR, involving severe NPDR and PDR. In accordance with ADA criteria assessing the necessity of prompt referral patients to a knowledgeable and experienced ophthalmologist for treatment and management [27], the classification we adopted is close to the clinical practice. As shown in our study, the STDR group exhibited a significantly higher value of baPWV than the NSTDR and NDR group, and the prevalence of STDR increased with increasing baPWV. Furthermore, degrees of baPWV were used in the multinominal logistic models adjusted for multiple confounders, demonstrating a positive relation of the highest tertile of baPWV to both NSTDR and STDR, with a further higher odds ratio in the latter group. In contrast, a study by Liu et al. exploring the degrees of baPWV and the severity of DR in 846 Chinese participants with T2DM [22] failed to obtain the significant association in the NPDR group, whereas the other study only documented the positive relationship with the presence of DR, but not the severity of DR [17]. The inconsistency might be due to a larger sample size in our study and relatively different classification of DR severity.

More importantly, in addition to the cross-sectional analysis, we conducted a follow-up analysis in 663 patients with T2DM to explore whether baseline baPWV was independently associated with the occurrence or progression of DR. To date, there were only two longitudinal studies with respect to the effect of AS on DR. First, a 6-year prospective study of 544 high cardiovascular risk Brazilian patients with T2DM has shown that aortic stiffness at baseline was not independently related to development/worsening of DR, while cfPWV during follow-up at the first year was a predictor for the development of DR but not progression of DR [36]. The discrepancy may be attributable to the race difference and stricter exclusion criteria in the present study, specifically with acute coronary syndrome, cerebrovascular disease, and peripheral arterial disease, which might influence the measurement of AS. Subgroup analysis from another study investigating the clustering of AS and microvascular complications of T2DM and their effects on the composite clinical endpoints found that PWV was correlated with neither the presence of microvascular complications at baseline nor its deterioration at follow-up [37]. The study defined microvascular complications as the presence of DR, diabetic kidney disease, and diabetic neuropathy or diabetic foot. Therefore, it could not sufficiently distinguish the separate effect of AS on DR. Inconsistently, the positive relationships between AS and DR as well as other components of microvascular complications, including diabetic kidney disease [38], peripheral neuropathy [39], and diabetic foot [40], have been well documented by previous studies. Our results revealed that increased AS at baseline independently predicted the development/progression of DR among Chinese patients with T2DM.

Recent prospective studies have demonstrated that baseline baPWV is independently associated with new onset of T2DM in general participants [41] and hypertensive patients [42], proposing the important role of AS in the development of T2DM. The underlying mechanism linking AS and T2DM and its microvascular complication, DR, remains to be elucidated. However, endothelial dysfunction, chronic inflammation, increased oxidative stress, and accumulation of AGEs have been previously proposed, as common pathogenic mechanisms, linking AS and progression of T2DM and its microvascular complications [43, 44].

The strength of the current study is that we confirmed the positive association of peripheral AS with the presence and the severity of DR, simultaneously, in a relatively larger cohort with more than 2,000 subjects, whereas the previous studies reported the relationship with respect to the presence and the severity of DR, respectively. Of note, we conducted a prospective cohort study combining cross-sectional and follow-up analyses and consistently demonstrated the predictive value of baPWV in the development/progression of DR. There are also several limitations in this study. First, patients with diabetic macular edema (DME) were not identified and included in the STDR group due to the lack of a gold standard for diagnosing DME, namely, optical coherence tomography measurements. Since DME can occur at any stage of DR, a missing diagnosis of DME may result in a bias to some extent. Second, the numbers of patients with newly onset or progression in DR were small due to a relatively short duration of follow-up. Further study with a longer follow-up is necessary. Additionally, we did not categorize the patients into those with occurrence and progression of DR, respectively, owing to small numbers of patients. Third, PWV measured from other segments, such as cfPWV, was not evaluated in the study. Therefore, similar results remain unclear concerning other PWV measurements.

## 5. Conclusion

In conclusion, we demonstrated that increased AS, indicated by a higher value of baPWV or an increased degree of baPWV, is associated with the presence and the severity of DR, both the NSTDR and STDR group. More importantly, baseline baPWV was an independent predictor of the development/worsening of DR. Taken together, these results suggested that worsening AS might be involved in the pathogenesis of DR. However, further research and follow-up studies with a longer duration are needed to validate the results and elucidate the possible mechanisms.

## Figures and Tables

**Table 1:** Independent associations of tertiles of baPWV with the presence and/or severity of DR.

	Crude		Adjusted	
	OR (95% CI)	*p* value	OR (95% CI)	*p* value
The presence of DR				
T1 baPWV	Ref.		Ref.	
T2 baPWV	1.50 (1.19, 1.88)	0.001^∗^	1.59 (1.21, 2.10)	0.001^∗^
T3 baPWV	2.68 (2.16, 3.35)	<0.001^∗^	2.73 (2.00, 3.74)	<0.001^∗^
The severity of DR				
NSTDR				
T1 baPWV	Ref.		Ref.	
T2 baPWV	1.50 (1.18, 1.90)	<0.001^∗^	1.62 (1.22, 2.15)	<0.001^∗^
T3 baPWV	2.43 (1.93, 3.06)	<0.001^∗^	2.58 (1.86, 3.58)	<0.001^∗^
STDR				
T1 baPWV	Ref.		Ref.	
T2 baPWV	1.47 (0.78, 2.78)	0.235	1.44 (0.71, 2.91)	0.310
T3 baPWV	4.87 (2.82, 8.43)	<0.001^∗^	3.87 (1.87, 8.02)	<0.001^∗^

Abbreviations: DR: diabetic retinopathy; NDR: non-DR; NSTDR: non-sight-threatening DR; STDR: sight-threatening DR. The fully adjusted logistic models were controlled for age, gender, BMI, current smoker, MAP, antihypertensive drugs, history of cardiovascular disease, duration of T2DM, HbA1c, TG, HDL-c, LDL-c, lipid-lowering drugs, eGFR, albuminuria, uria acid, ALT, and AST. ∗ presents a *p* value reaching statistical significance.

**Table 2:** Clinical characteristics of patients in the follow-up analysis divided by tertiles of baPWV.

	T1 baPWV	T2 baPWV	T3 baPWV	*p* value
	(*n* = 219)	(*n* = 218)	(*n* = 226)	
Age (years)	43.6 ± 11.1	51.6 ± 10.3	58.5 ± 9.0	<0.001^∗^
Gender (male/female)	148/71	123/95	115/111	0.001^∗^
Body mass index (kg/m^2^)	27.1 ± 4.4	26.7 ± 4.1	26.4 ± 3.4	0.202
Current smoker (*n*, %)	65 (29.7%)	56 (25.7%)	41 (18.2%)	0.017^∗^
Alcohol consumption (*n*, %)	35 (16.0%)	37 (17.0%)	41 (18.2%)	0.821
Hypertension (*n*, %)	64 (29.2%)	125 (57.3%)	171 (75.7%)	<0.001^∗^
Systolic BP (mmHg)	122.1 ± 12.6	131.6 ± 15.4	140.2 ± 17.0	<0.001^∗^
Diastolic BP (mmHg)	77.2 ± 10.0	80.1 ± 10.8	81.1 ± 12.5	0.001^∗^
Antihypertensive drugs	39 (17.8%)	76 (34.9%)	114 (50.9%)	<0.001^∗^
History of CVD (*n*, %)	24 (11.0%)	27 (12.4%)	35 (15.5%)	0.382
Duration of T2DM (months)	36.0 (1.0, 76.5)	63.5 (15.8, 148.0)	109.0 (38.00, 167.0)	<0.001^∗^
HbA1c (%)	9.0 ± 2.5	8.4 ± 2.1	8.6 ± 1.9	0.020^∗^
Fasting serum glucose (mmol/L)	9.6 ± 3.9	9.2 ± 4.0	9.9 ± 4.1	0.208
Fasting C-peptide (ng/mL)	2.14 ± 1.02	2.13 ± 1.14	2.31 ± 1.10	0.149
Hyperlipidemia (*n*, %)	154 (70.3%)	146 (67.0%)	167 (73.9%)	0.355
Triglycerides (mmol/L)	1.65 (1.10, 2.52)	1.54 (1.06, 2.50)	1.59 (1.09, 2.33)	0.465
Total cholesterol (mmol/L)	4.80 ± 1.12	4.81 ± 1.38	4.79 ± 1.08	0.983
HDL-c (mmol/L)	1.16 ± 0.28	1.19 ± 0.25	1.20 ± 0.28	0.237
LDL-c (mmol/L)	3.06 ± 0.86	3.04 ± 0.89	3.03 ± 0.85	0.944
Drugs for hyperlipidemia	58 (26.5%)	60 (27.5%)	72 (31.9%)	0.477
ALT (U/L)	32.8 ± 25.6	27.2 ± 19.7	28.0 ± 19.6	0.016^∗^
AST (U/L)	21.9 ± 11.9	20.0 ± 9.0	21.3 ± 12.0	0.209
Creatinine (*μ*mol/L)	68.0 ± 14.0	67.2 ± 17.3	68.8 ± 15.4	0.552
Urea nitrogen (mmol/L)	4.78 ± 1.29	4.99 ± 1.66	5.51 ± 1.47	<0.001^∗^
eGFR (mL/min/1.73 m^2^)	107.4 ± 16.3	100.8 ± 16.7	93.4 ± 14.5	<0.001^∗^
Uric acid (*μ*mol/L)	334.6 ± 97.6	324.6 ± 86.3	326.9 ± 83.6	0.479
Albuminuria				0.002^∗^
Normoalbuminuria (*n*, %)	169 (80.1%)	159 (76.4%)	139 (63.2%)	
Microalbuminuria (*n*, %)	35 (16.6%)	42 (20.2%)	70 (31.8%)	
Macroalbuminuria (*n*, %)	7 (3.3%)	7 (3.4%)	11 (5.0%)	
baPWV (m/s)	12.29 ± 0.94	14.74 ± 0.65	18.43 ± 2.25	<0.001^∗^
Newly onset or progression of DR (*n*, %)	26 (11.9%)	41 (18.7%)	44 (19.6%)	0.052

Continuous variables are shown as mean ± S.D. for parametric data or median (interquartile ranges) for nonparametric data. Categorical variables are expressed as numbers and percentages. Abbreviations: BP: blood pressure; CVD: cardiovascular disease; HbA1c: hemoglobin A1c; HDL-c: high-density lipoprotein cholesterol; LDL-c: low-density lipoprotein cholesterol; ALT: alanine aminotransferase; AST: aspartate aminotransferase; eGFR: estimated glomerular filtration rate; baPWV: brachial-ankle pulse wave velocity; DR: diabetic retinopathy. ∗ indicates the difference among the three groups reaching significance.

**Table 3:** Independent association between degrees of baPWV and new onset/progression of DR revealed by Cox proportional-hazards regression analysis.

	T2 PWV		T3 PWV	
	HR (95% CI)	*p* value	HR (95% CI)	*p* value
Crude	1.84 (1.12, 3.02)	0.016^∗^	2.07 (1.28, 3.36)	0.003^∗^
Model 1	2.23 (1.33, 3.75)	0.002^∗^	3.04 (1.72, 5.37)	<0.001^∗^
Model 2	2.29 (1.33, 3.95)	0.003^∗^	2.84 (1.55, 5.18)	<0.001^∗^
Model 3	2.24 (1.25, 4.03)	0.007^∗^	2.90 (1.49, 5.64)	0.002^∗^

Model 1 was adjusted for age and gender; model 2 was controlled for age, gender, duration of T2DM, and HbA1c; model 3 was further adjusted for MAP, usage of hypertensive medication, hyperlipidemia, eGFR, albuminuria, and uric acid based on model 2. ∗ presents a statistically significant *p* value.

## Data Availability

The data used to support the findings of this study are available from the corresponding author upon request.

## Supplementary Materials

Supplementary Materials Supplemental Table 1: clinical characteristics of all patients with T2DM at baseline stratified by tertiles of baPWV.
