# Supplementary material for: Increased Arterial Stiffness as a Predictor for Onset and Progression of Diabetic Retinopathy in Type 2 Diabetes Mellitus
Source: J Diabetes Res. 2021 Sep 23;2021:9124656. doi: 10.1155/2021/9124656 (PMC8486550; doi:10.1155/2021/9124656)
Supplement: Supplementary Materials — Supplemental Table 1: clinical characteristics of all patients with T2DM at baseline stratified by tertiles of baPWV. [file 9124656.f1.docx]

**Supplemental Table 1. Clinical characteristics of all patients with T2DM at baseline stratified by tertiles of baPWV**

|  | T1 baPWV | T2 baPWV | T3 baPWV | *p* value |
| --- | --- | --- | --- | --- |
|  | (n=811) | (n=821) | (n=841) |  |
| Age (years) | 43.5 ± 11.1 | 51.3 ± 10.8 | 59.3 ± 9.3 | <0.001* |
| Gender (male/female) | 528/283 | 478/343 | 418/423 | <0.001* |
| Body Mass Index (kg/m^2^) | 27.1 ± 4.4 | 26.9 ± 4.0 | 26.3 ± 3.6 | <0.001* |
| Current smoker (n, %) | 271 (33.4%) | 232 (28.2%) | 167 (19.8%) | <0.001* |
| Alcohol consumption (n, %) | 108 (13.3%) | 126 (15.4%) | 117 (13.9%) | 0.483 |
| Hypertension (n, %) | 271 (33.4 %) | 461 (56.2%) | 625 (74.3%) | <0.001* |
| Systolic BP (mmHg) | 124.1 ± 14.2 | 132.3 ± 15.4 | 140.0 ± 18.5 | <0.001* |
| Diastolic BP (mmHg) | 77.7 ± 10.5 | 81.0 ± 11.6 | 81.3 ± 12.3 | <0.001* |
| Anti-hypertensive drugs | 149 (18.4 %) | 273 (33.3%) | 423 (50.4%) | <0.001* |
| History of CVD (n, %) | 69 (8.5%) | 86 (10.5%) | 138 (16.4%) | <0.001* |
| Duration of T2DM (months) | 27.0 (1.0, 84.0) | 59.0 (12.0, 130.0) | 119.0 (38.0, 181.0) | <0.001* |
| HbA1c (%) | 9.1 ± 2.5 | 8.7 ± 2.2 | 8.9 ± 2.0 | 0.001* |
| Fasting serum glucose (mmol/L) | 9.5 ± 4.4 | 9.3 ± 4.0 | 9.6 ± 4.4 | 0.443 |
| Fasting C-Peptide (ng/mL) | 2.2 ± 1.2 | 2.2 ± 1.1 | 2.2 ± 1.3 | 0.486 |
| Hyperlipidemia (n, %) | 557 (68.7%) | 585 (71.3%) | 596 (70.9%) | 0.466 |
| Triglycerides (mmol/L) | 1.59 (1.07, 2.50) | 1.58 (1.12, 2.50) | 1.48 (1.02, 2.21) | 0.006* |
| Total cholesterol (mmol/L) | 4.85 ± 1.26 | 4.93 ± 1.31 | 4.80 ± 1.23 | 0.123 |
| HDL-c (mmol/L) | 1.15 ± 0.29 | 1.18 ± 0.27 | 1.20 ± 0.28 | <0.001* |
| LDL-c (mmol/L) | 3.10 ± 0.89 | 3.16 ± 0.92 | 3.04 ± 0.95 | 0.048* |
| Drugs for hyperlipidemia | 176 (21.7 %) | 190 (23.2%) | 248 (29.5%) | <0.001* |
| ALT (U/L) | 34.6 ± 30.9 | 29.6 ± 27.3 | 25.6 ± 19.0 | <0.001* |
| AST (U/L) | 23.5 ± 16.2 | 21.7 ± 14.8 | 20.9 ± 11.9 | 0.001* |
| Creatinine (μmol/L) | 66.0 ± 14.6 | 67.7 ± 16.9 | 68.8 ± 18.1 | 0.003* |
| Urea nitrogen (mmol/L) | 4.7 ± 1.4 | 5.1 ± 1.5 | 5.5 ± 1.7 | <0.001* |
| eGFR (mL/min/1. 73m^2^) | 108.8 ± 16.1 | 100.4 ± 16.7 | 92.6 ± 15.8 | <0.001* |
| Uric acid (μmol/L) | 329.8 ± 95.6 | 331.4 ± 91.4 | 317.8 ± 84.1 | 0.004* |
| Albuminuria |  |  |  | <0.001* |
| Normoalbuminuria (n, %) | 615 (79.3%) | 574 (73.2%) | 503 (62.0%) |  |
| Microalbuminuria (n, %) | 141 (18.2%) | 177 (22.6%) | 232 (28.6%) |  |
| Macroalbuminuria (n, %) | 20 (2.6%) | 33 (4.2%) | 77 (9.5%) |  |
| baPWV (m/s) | 12.28 ± 1.03 | 14.86 ± 0.71 | 18.76 ± 2.32 | <0.001* |
| Presence of DR (n, %) | 165 (20.4 %) | 227 (27.7%) | 342 (40.7%) | <0.001* |
| Severity of DR |  |  |  | <0.001* |
| NSTDR | 148 (18.3 %) | 204 (24.9%) | 278 (33.1%) |  |
| Mild NPDR | 123 (15.2 %) | 166 (20.2%) | 195 (23.2%) |  |
| Moderate NPDR | 25 (3.1 %) | 38 (4.6%) | 83 (9.9%) |  |
| STDR | 17 (2.1 %) | 23 (2.8%) | 64 (7.6%) |  |
| Severe NPDR | 7 (0.9 %) | 13 (1.6%) | 41 (4.9%) |  |
| PDR | 10 (1.2 %) | 10 (1.2 %) | 23 (2.7%) |  |

Continuous variables are shown as mean ± S.D. for parametric data or median (interquartile ranges) for non-parametric data. Categorical variables are expressed as numbers and percentages. Abbreviations: BP, blood pressure; CVD, cardiovascular disease; HbA1c, hemoglobin A1c; HDL-c, high-density lipoprotein cholesterol; LDL-c, low-density lipoprotein cholesterol; ALT, Alanine aminotransferase; AST, Aspartate aminotransferase; eGFR, estimated Glomerular Filtration Rate; baPWV, brachial-ankle pulse wave velocity; DR, diabetic retinopathy; NSTDR, non sight-threatening DR; NPDR, non-proliferative DR; STDR, sight-threatening DR; PDR, proliferative DR. * indicates the difference among three groups reaching significance.
